# Case Report: Passive Handstand Promotes Cerebrovascular Elasticity Training and Helps Delay the Signs of Aging: A 40-Year Follow-Up Investigation

**DOI:** 10.3389/fmed.2022.752076

**Published:** 2022-04-26

**Authors:** Haonan Liu, Qian Xu, Xin Xiang, Danan Liu, Shengyong Si, Lan Wang, Ying Lv, Yidong Liao, Hua Yang

**Affiliations:** ^1^Department of Neurosurgery, The Affiliated Hospital of Guizhou Medical University, Guiyang, China; ^2^Department of Neurology, Shanghai Ninth People’s Hospital, Shanghai Jiao Tong University School of Medicine, Shanghai, China; ^3^Institute of Medical Science, Guizhou Medical University, Guiyang, China

**Keywords:** passive handstand exercise, cerebrovascular elasticity, aging, gravity, cervical spondylosis

## Abstract

**Background:**

There are no long-term (>10 years) follow-up evaluations of the effects of handstand exercise or studies on the use of equipment for passive handstand exercise.

**Objective:**

To report a 40-year follow-up investigation of a Chinese man who has been practicing passive handstand for 40 years.

**Design:**

This observational investigation was conducted in Guizhou Province, China.

**Participant:**

A (currently) 66-year-old Chinese man who had been practicing passive handstand exercise for 40 years was followed up.

**Interventions:**

Physical and auxiliary examinations were carried out to determine the effects of long-term passive handstand exercise on the human body.

**Main Measures:**

The participant’s cerebrovascular, spinal health, mental health, and visual acuity as well as the presence of facial aging were examined.

**Key Results:**

His cerebral vessels were healthy, he appeared younger than his peers, his cervical spondylosis improved, and his mental state and cognitive function were good.

**Conclusion:**

Long-term passive handstand exercise can promote cerebrovascular elasticity training and delay signs of aging. We recommend promoting this passive handstand exercise to the public.

## Introduction

Humans have long been accustomed to walking on their feet, bearing the effect of gravity on the body from head to toe (1). The presence of gravity increases the resistance of the heart to pump blood to organs superior to the heart. Besides, a previous study (2) showed that exposure to simulated microgravity induced cellular senescence through increased oxidant stress. Standing on the head is, thus, not a typical posture but is of interest, because, in this position, the normal erect posture, which man has acquired in the course of evolution, is totally reversed. The metabolic cost and cardiovascular and respiratory responses to this posture were reported previously (3–5). Vital capacity is at its maximum when an individual is standing erect and at its minimum when an individual is in the handstand position (3). The systolic blood pressure in handstand is approximately 20 mmHg higher than that in the erect position (4). The handstand position consumes more energy than standing erect (5). However, the long-term effect of handstand posture on human health is unclear.

We identified very few studies (3–8) related to handstand or headstands, with most of them being not related to the effects of handstand exercise on health. To the best of our knowledge, no studies have performed long-term (>10 years) follow-up evaluations of the effects of handstand exercise or studies on the use of equipment for passive handstand exercise.

We herein report the findings of a 40-year follow-up investigation of a Chinese man who had been practicing passive handstand exercise for 40 years, with the aim of determining the effects of long-term passive handstand exercise on the human body.

## Methods

This study was approved by the Ethics Committee of Guizhou Medical University [approval number 2020 (210)]; the approval was obtained in 2020, as this committee had not been established 40 years ago. The participant provided written informed consent and volunteered to participate in the study.

### Participant

The participant is a (currently) 66-year-old Chinese Han man born and raised in Guizhou, China. He is married, well-educated, and retired (used to work as a teacher and civil servant). He does not smoke, does not take drugs, and drinks alcohol in moderation (<140 ml of ethanol/week). We investigated his daily habits, eating preferences, physical activities, etc., and there was nothing special except the handstand concerned in this study. The participant was healthy 40 years ago, and he did handstand exercise because he wanted to stay fit.

### Device

**FIGURE 1:**
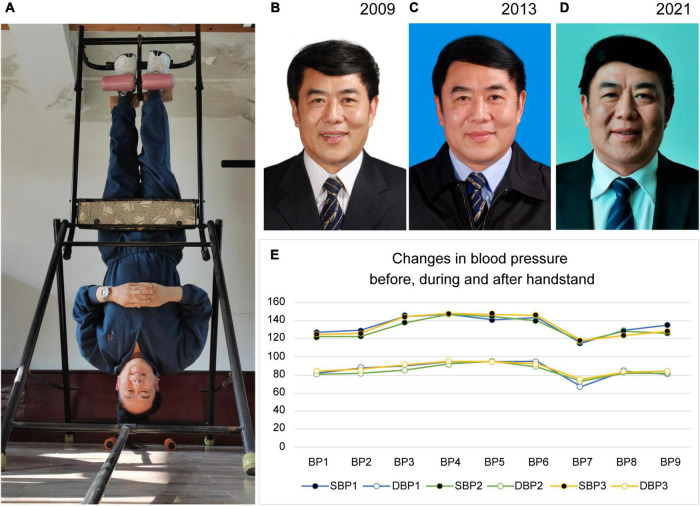
Changes in appearance over 10 years and changes in blood pressure during device use. **(A)** The method of passive handstand exercise. When using the device, the feet are placed in the foot retainer, and the support frame is held to lower the body upside down. **(B–D)** Participant’s appearance in the 2009, 2013, and 2021 assessments. **(E)** Blood pressure changes (recorded every 2 min) before, during, and after a handstand are as follows: rest (BP1), moment before handstand (BP2), handstand for 2 min (BP3), handstand for 4 min (BP4), handstand for 6 min (BP5), handstand for 8 min (BP6), end of handstand exercise and return to normal position (BP7), rest at 2 min after the handstand (BP8), and rest at 4 min after the handstand (BP9). SBP, systolic blood pressure; DBP, diastolic blood pressure. Permission to utilize photographs was obtained.

The participant designed the passive handstand device. The device includes a V-shaped support frame, a high bracket for handstand, and a foot retainer (Figure 1). When he was young, he used the device to exercise for approximately 30 min at a time, but because of his busy schedule and many business trips, he was sometimes unable to exercise on time. When he retired at age 60, he persisted in doing handstand exercises once daily for only 10 min. When using the device, he did not experience discomfort.

### Evaluations

We quantified health through physical and auxiliary examinations, including imaging studies to evaluate the participant’s cerebrovascular, spinal health, facial aging, mental health, and visual acuity. Assessments were performed every 10 years. In the first 20 years of follow-up, due to the underdeveloped medical technology and the lack of auxiliary examination at that time, physical examination was mainly used.

#### Physical and Auxiliary Examinations

In 2021, spinal health evaluation comprised physical examination at each follow-up and magnetic resonance imaging. At the 20-year follow-up, we examined the participant’s cervical spine using radiography. Cerebrovascular assessment consisted of computed tomography angiography and magnetic resonance angiography. We compared his current cerebrovascular condition (assessed by magnetic resonance angiography) with that 10 years prior (assessed by computed tomography angiography) to confirm the effect of long-term handstand suspension on cerebrovascular elasticity. We also evaluated his cerebrovascular health by measuring his blood pressure before, during, and after using the device.

#### Facial Aging

We took pictures of the participant and compared them with those taken more than a decade prior to assess the rate of facial aging. Facial aging was assessed according to the severity of eyelid pouches and facial wrinkles. The severity of eyelid pouches was categorized as no pouches, mild pouches (slight herniation of the lower orbital septum fat accompanied by slight relaxation of the lower eyelid skin), moderate pouches (moderate herniation of the lower orbital septum fat accompanied by moderate relaxation of the lower eyelid skin), and severe pouches (severe herniation of the lower orbital septum fat or severe relaxation of the lower eyelid skin). The severity of facial wrinkles was evaluated using the Wrinkle Severity Rating Scale (9).

#### Visual Acuity

Since 1991, the visual acuity of this man was measured according to the Early Treatment of Diabetic Retinopathy Study protocol using a LogMAR chart (10). The test results of the better eye were expressed in logarithmic units ranging from 1 (20/200) to −0⋅3 (20/10), with lower values indicating better vision.

#### Mental Health

Moreover, since 1991, we have assessed his mental health and cognitive function using the Mini Mental State Examination scale (11).

### Role of the Funding Source

The sponsor (Science and Technology Innovation Talent Team Project of Guizhou Province, China) provided financial support in the collection and interpretation of data.

### Patient and Public Involvement

The patient and the public were involved in the design, reporting, or dissemination plans of our research. The participant made some suggestions for the design of our research.

## Results

### General Characteristics

The method of using the device, changes in appearance during the past 10 years, and changes in blood pressure before, during, and after a handstand are shown in Figure 1. The participant is currently 66 years old, but he does not appear to be at this age in terms of physical characteristics, as he remained relatively youthful and energetic. During the 40 years of follow-up, every physical examination showed that he was in good health. He has no hypertension, diabetes mellitus, coronary heart disease, cerebrovascular disease, chronic obstructive pulmonary disease, benign prostatic hyperplasia, hemorrhoids, hair loss, vision loss, insomnia, or mental illness.

### Cerebrovascular Health

**FIGURE 2:**
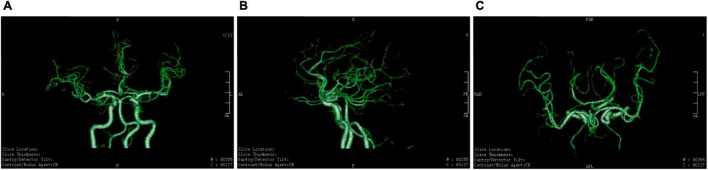
Cerebral vessels in 2011. **(A–C)** Head computed tomography angiography findings in 2011. His cerebral vessels ran normally, and there was no stenosis of the lumen.

**FIGURE 3:**
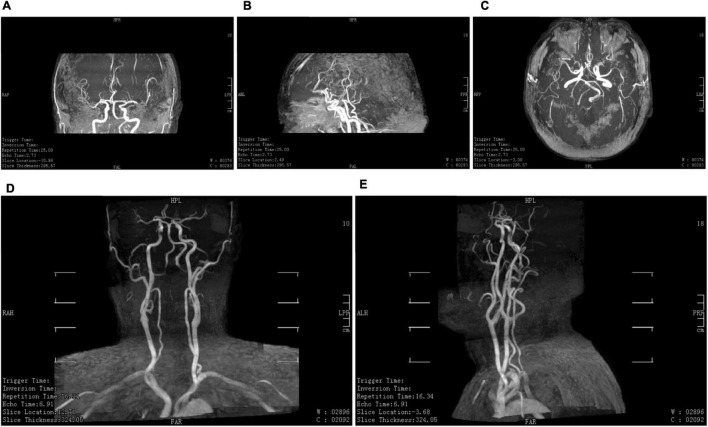
Cerebral vessels in 2020. **(A–E)** Head and neck magnetic resonance angiography in 2020. The results of his cerebrovascular tests in 2020 were similar to those in 2011.

His cerebrovascular health is indicated by cerebrovascular images in Figures 2, 3. Ten years ago, he had good cerebral vessels. In the latest follow-up, we observed no apparent aging of the blood vessels, as his cerebral vessels ran normally and there was no stenosis of the lumen. His blood pressure was in the normal range before the exercise, immediately increased by approximately 20 mmHg during the exercise, and remained basically higher for 8 min than that in the upright position. After the handstand, he returned to the upright position, and his blood pressure dropped rapidly, which was lower than the normal level, but it then immediately normalized. These findings indicate good cerebral vascular elasticity.

### Spinal Health

**FIGURE 4:**
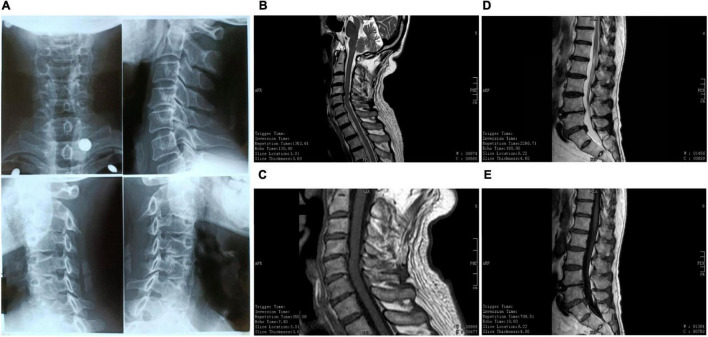
Spine. **(A)** Cervical X-ray examination in 2002. At that time, he was diagnosed with cervical degeneration and narrowing of the intervertebral foramen and intervertebral space. **(B,C)** Cervical magnetic resonance imaging (MRI) scan in 2020. **(D,E)** Lumbar MRI in 2020.

His spinal health is shown in Figure 4. He was diagnosed with cervical spondylosis 20 years ago due to excessive desk work. Despite a slight protrusion of the cervical and lumbar vertebrae, he is currently free of symptoms and is able to move freely without any neck or lower back pain or numbness in the hands or feet.

### Facial Aging

Few wrinkles and bags are visible under the eyes. The blood supply to the head is well supplied by the long training in handstands. He achieved a Wrinkle Severity Rating Scale grade of 3, indicating moderate wrinkles and moderate eyelid pouches.

### Visual Acuity

His visual acuity was −0.1 in both eyes in the 2001, 2011, and 2021 assessments.

### Mental Health

His Mini Mental State Examination score was 30 in the 1991, 2001, 2011, and 2021 assessments. In the recent follow-up, his mental state and cognitive function were normal, and he was able to speak clearly and answer fluently.

## Discussion

To determine the effects of long-term passive handstand exercise on the human body, we conducted a 40-year follow-up investigation of a Chinese man who practiced passive handstand. During the 40 years, his cerebral vessels remained healthy, he had a younger appearance relative to his peers, his cervical spondylosis improved, and his mental state and cognitive function were good. This indicates that long-term passive handstand exercise can promote cerebrovascular elasticity training and delay signs of aging.

As life expectancy increases and fertility rates decline, most countries become “aging societies,” with more people aged > 65 years than aged < 15 years (12). China is now in a transitional period in which health is recognized as the centerpiece of sustainable development, as highlighted in the Healthy China 2030 plan adopted in 2016 (13).

Stroke is the second leading cause of death globally and the leading cause of death in China, where 20% of the world’s population resides (14). Cerebrovascular elasticity is closely related to intracranial vascular health (15). Aging is the main risk factor contributing to vascular dysfunction and vascular disease progression. Stem cell and gene therapies may restore impaired vascular cell function, reverse vascular aging, and prolong an individual’s lifespan (16). Our participant’s cerebral vessels were very healthy. His blood pressure remained normal before the handstand exercise, increased during the handstand, and quickly returned to normal after the handstand, indicating good vascular elasticity. We, thus, believe that passive handstand can promote cerebrovascular elasticity training, which is of great benefit in the prevention and treatment of cerebral atherosclerosis and brain ischemia. Sir William Osler (as cited in a previous study) (17) stated, “Longevity is a vascular question which has been well expressed in the axiom that man is only as old as his arteries.” Our participant also believes in this view. Both his parents died from cerebrovascular disease, but his cerebral vessels were very healthy, which may reverse the family history to some extent.

Cerebrovascular functions decline during normal aging, with pronounced effects observed in patients with Alzheimer’s disease (18). In terms of cognitive function, our participant has a sharp mind and is still teaching doctoral students. He works for 5 h every morning, but his mind is clear, and he does not get tired quickly. Thus, persistent handstand exercise may also play an important role in improving brain response speed and delaying onset of Alzheimer’s disease symptoms.

Previous case reports have highlighted the association of the handstand position with intraocular pressure and eye disease (6–8). A previous study reported a uniform twofold increase in the intraocular pressure during handstand (6), but it did not demonstrate a higher prevalence of ocular hypertension in handstand nor did the risk factors contributing to glaucoma show any correlation with the magnitude of intraocular pressure increase during the posture. A case of Valsalva retinopathy probably induced by straining that occurred following a handstand was previously reported (7). Moreover, progressive glaucomatous optic neuropathy and visual field loss were reported to have occurred in a patient who practiced the Sirsasana (headstand) yoga posture on a daily basis for many years (8). Our participant has been practicing handstand exercise for a long time, and his good eyesight has been well maintained. Regarding his appearance, he appears younger than his peers, with moderate eyelid pouches and no color spots, hair loss, or calf edema. Thus, handstand exercise may delay the signs of aging and promote eye health.

Many yogis and exercisers already practice active handstands. However, for non-yogis, particularly the older ones, active handstands are difficult. The device designed by our participant makes passive handstands easy to perform. However, whether passive handstand is suitable for people with cerebrovascular disease or those with high risk factors for cerebrovascular disease warrants further study.

Based on this 40-year follow-up investigation and previous literature, we believe that passive handstand promotes cerebrovascular elasticity training, thereby improving the functional reserve of intracranial blood vessels, promoting blood supply to the head, rejuvenating the face, and delaying signs of aging. It can be used as a rehabilitation treatment for individuals with disorders in the cervical and lumbar vertebrae, and it can improve mental health and cognitive function. However, more studies with a larger sample size are required to understand the true relationship between passive handstand and its health benefits.

In conclusion, passive handstand is easier to popularize than active handstands. We recommend 30 min of passive handstand exercise daily before age 40 years, 20 min at age 40–60 years, and 10 min at age > 60 years. In order to prevent danger, it is best to have someone present when doing this exercise. If this exercise can be promoted globally, it will further the development of preventive medicine and the sports health industry.

## Data Availability Statement

The raw data supporting the conclusions of this article will be made available by the authors, without undue reservation.

## Ethics Statement

The studies involving human participants were reviewed and approved by the Ethics Committee of Guizhou Medical University. The patients/participants provided their written informed consent to participate in this study. Written informed consent was obtained from the individual(s) for the publication of any potentially identifiable images or data included in this article.

## Author Contributions

HL contributed to the retrieval and review of previous literatures, collection and assembly of data, connection with the participant, data analysis and interpretation, and manuscript writing. QX contributed to collection and assembly of data, data analysis and interpretation, and revision of the manuscript. XX, DL, and LW contributed to the follow-up of the participant. SS and YLi contributed to the retrieval of previous literatures and data analysis. YLv contributed to the collection and assembly of data, connection with the medical record department of the hospital. HY contributed to conception, design, provision of study materials, connection with the participant, provision of study materials, data analysis and interpretation, and attests that all listed authors meet the authorship criteria and that no others meeting the criteria have been omitted. HL, QX, and HY have had access to, and verified, the data in this manuscript. All authors contributed to the final approval of manuscript and agreed to be accountable for the content of the work.

## Conflict of Interest

The authors declare that the research was conducted in the absence of any commercial or financial relationships that could be construed as a potential conflict of interest.

## Publisher’s Note

All claims expressed in this article are solely those of the authors and do not necessarily represent those of their affiliated organizations, or those of the publisher, the editors and the reviewers. Any product that may be evaluated in this article, or claim that may be made by its manufacturer, is not guaranteed or endorsed by the publisher.
